# Anti-*Toxoplasma gondii* Antibodies in European Residents: A Systematic Review and Meta-Analysis of Studies Published between 2000 and 2020

**DOI:** 10.3390/pathogens12121430

**Published:** 2023-12-08

**Authors:** Rafael Calero-Bernal, Solange María Gennari, Santiago Cano, Martha Ynés Salas-Fajardo, Arantxa Ríos, Gema Álvarez-García, Luis Miguel Ortega-Mora

**Affiliations:** 1 SALUVET, Animal Health Department, Complutense University of Madrid, 28040 Madrid, Spain; marthays@ucm.es (M.Y.S.-F.); arantxarios@gmail.com (A.R.); gemaga@ucm.es (G.Á.-G.); luis.ortega@ucm.es (L.M.O.-M.); 2 PhD Program in One Health, Faculty of Veterinary Medicine, University of Santo Amaro, São Paulo 04829-300, SP, Brazil; sgennari@usp.br; 3 Faculty of Veterinary Medicine, University of São Paulo, São Paulo 05508-270, SP, Brazil; 4 Computing Services, Research Support Center, Complutense University of Madrid, 28040 Madrid, Spain; scano@ucm.es

**Keywords:** *Toxoplasma gondii*, human, Europe, seroprevalence, risk factors, meta-analysis

## Abstract

Toxoplasmosis has a major impact on animal and public health. Information regarding the seroprevalence of human *Toxoplasma gondii* infections from a European perspective has not yet been compiled to date. Thus, the present review summarized available resident data from the period 2000–2020. The overall seroprevalence of anti-*T. gondii* IgG was 32.1%, with great variability between countries (*n* = 30). The subgroup analysis identified different pooled prevalence data depending on the geographic area (*p* < 0.0001), target population (*p* = 0.0147), and serological diagnosis assays used (*p* = 0.0059). A high heterogeneity (I^2^ = 100%, *p* < 0.001; Q = 3.5e+05, d.f. = 135, *p* < 0.001) and degree of publication bias (Egger’s test = 6.14, *p* < 0.001) were observed among the 134 studies considered. The occurrence of anti-*T. gondii* IgM, which was reported in 64.7% of studies, reached a pooled seroprevalence of 0.6%. In addition, among the eight main risk factors identified, “contact with soil”, “consumption of undercooked beef”, and “intake of unwashed vegetables” were the most significantly associated with infections. The fact that one-third of the European population has been exposed to *T. gondii* justifies extra efforts to harmonize surveillance systems and develop additional risk-factor analyses based on detailed source attribution assessment.

## 1. Introduction

*Toxoplasma gondii* is an apicomplexan intracellular parasite capable of infecting almost all homoeothermic animals, including humans [1]. *Toxoplasma gondii* is characteristically opportunistic, and may be especially harmful in immunocompromised patients (HIV-positive, solid organ transplant recipients, etc.), but also when primary infections occur during pregnancy; congenital infections may lead to important reproductive disorders from abortions, fetal malformations, or diverse mental-retardation sequelae in children. A global disease burden estimation study [2] identified an association between the occurrence of latent toxoplasmosis and specific disease burden in 88 countries, and indeed such correlations between the presence of anti-*Toxoplasma* antibodies and age-standardized disability adjusted life years (DALY) explained 23% of the variability in disease burden in 29 European countries.

Comprehensive literature reviews of the status of *T. gondii* infections (a major zoonosis) in domestic and wild animals in Europe have been carried out [3,4]; nevertheless, regarding human populations, only one paper [5] reviewed the epidemiological situation focused on the Balkan countries (southeast Europe), and therefore no studies have been carried out from a pan-European perspective to date. Two studies analyzed the current surveillance schemes set up in Austria, France, and the USA, and highlighted the need to harmonize diagnosis and monitoring systems in most countries [6,7].

Despite the incidence of congenital toxoplasmosis in Europe (5.8 cases per 100,000 live births), which is ranked among the top causes of disease burden in EU/EEA when evaluating the disability-adjusted life years parameter [8,9], well-structured investigations concluded that most *T. gondii*-infections in the EU had a food-borne origin [10]. In this regard, a pioneer multicenter case–control study aiming at the identification of sources of *Toxoplasma*-acute infections in pregnant women in Europe [11] suggested eating undercooked lamb, beef, or game meat, contact with soil, and traveling outside Europe, USA, and Canada as the major risk factors. However, remarkably, contact with cats was not identified as a risk factor. In agreement, a later meta-analysis that was focused on worldwide consumption habits [12] identified the intake of raw/undercooked beef and lamb meat as risk factors significantly associated with acute *T. gondii* infections in humans. In spite of this, the authors exposed the limitations due to the low number of case–control studies available. Recently, a case–control study identified consumption of meat of large game animals and poor hand hygiene as key risk factors for acute toxoplasmosis in the Netherlands [13]. The complex epidemiology of *T. gondii* is responsible for the high number of risk factors of infection reported worldwide (reviewed in [14]). Currently, some habits and trends may have changed in the European population. In recent years, an increase in *T. gondii* infection cases has been clearly associated with fresh vegetable consumption [15], and it has been demonstrated that the environmental route of infection may involve a wide set of potential sources [16]. Accordingly, new studies approaching the source attribution of *T. gondii* infections have resulted in major interest [17].

As commented above, no review paper focused on seroprevalence data on European human populations has been carried out yet; therefore, the present article aimed to systematically review and carry out a further meta-analysis of the available literature regarding two important aspects in observational studies published from 2000 to 2020: seroprevalence (IgG and IgM) data and the most suitable risk factors involved in *T. gondii* infections in European residents.

## 2. Materials and Methods

**Figure 1:**
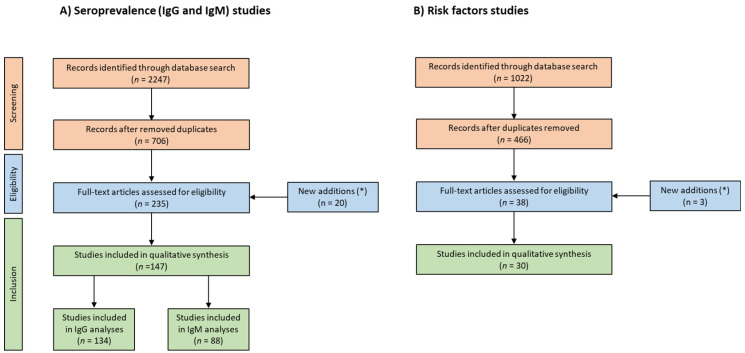
Flowchart describing the study design process. Description of the study search and selection for publications related to (**A**) seroprevalence of IgG and IgM anti-*T. gondii* antibodies, and (**B**) risk factors analyses. (*) A secondary search was carried out based on references included in articles examined for eligibility.

The present study was carried out according to the PRISMA guidelines (Preferred Reporting Items for Systematic Reviews and Meta-Analyses) [18], and the PRISMA checklist is available in Supplementary Table S1. The performance workflow followed in the present study is shown in Figure 1. 

Literature published between January 2000 and December 2020 was searched in electronic databases (PubMed, Science Direct, Scopus, WoS and SCIelo) using medical subject headings (MeSH) terms (*Toxoplasma gondii*, toxoplasmosis, seroprevalence, human, Europe); selection (eligibility criteria: serosurveys [excluding review papers; case–control studies were only considered for the risk factors study/analyses], years 2000 to 2020, continental Europe, any language); and further critical review and a meta-analysis were carried out as previously proposed [19]. Initially, abstracts were revised for study screening and further data extraction; the whole process was carried out by two investigators. Additional references cited in the bibliography of the evaluated papers were considered eligible. Papers in languages other than English (to note: Croatian, French, German, Icelandic, Italian, Polish, Portuguese, Serbian, Spanish, and Russian) were taken into consideration. 

From each eligible study dealing with anti-*T. gondii* IgG and IgM data or risk factors analysis, detailed information on study area, year/data of sampling, target population, diagnostic method, sample size, and number of positive samples was collected. In addition, data on significant risk factor conditions (odds ratio, relative risk, and significance) were gathered in those specific studies (Supplementary Table S2). Regarding data analysis, for the evaluation of pooled estimates (detection rates reported in each study), a restricted maximum likelihood method with a random effects model was used [20]. Study bias and heterogeneity at the study level were calculated using Egger’s test, and Cochran’s Q test and inverse variance index (I^2^), respectively [20,21,22]. Alpha was set at 0.05.

Statistical analyses were performed using STATA 15.0 software (StataCorp, Bryan, TX, USA).

## 3. Results

### 3.1. Seroprevalence of Anti-T. gondii IgG in European Residents

A total of 136 published studies on *T. gondii* IgG seroprevalence in humans in Europe, published between 2000 and 2020, with 30 countries represented, were eligible and included in this meta-analysis (Table 1). Europe was divided into 4 regions: North, with 7 countries (Estonia, Finland, Iceland, Ireland, Norway, Sweden, and United Kingdom) and 51 published studies; West, with 6 countries (Austria, Belgium, France, Germany, Netherlands, and Switzerland) and 47 studies; East, with 6 countries (Czech Republic, Hungary, Poland, Romania, Russia, and Slovakia) and 18 publications; and finally South, with 11 countries (Albania, Bosnia and Herzegovina, Croatia, Cyprus, Greece, Italy, North Macedonia, Portugal, Serbia, Slovenia, and Spain) and 20 studies. For the analysis of IgG seroprevalence, the samples were obtained from the following: general population (*n* = 38 studies), pregnant women or women of childbearing age (*n* = 58), and others (*n* = 47), and the diagnostic methods that were used were based on commercial tests (*n* = 101 studies), in-house tests (*n* = 15), and a combination of both (*n* = 8) (Table 2).

**Figure 2:**
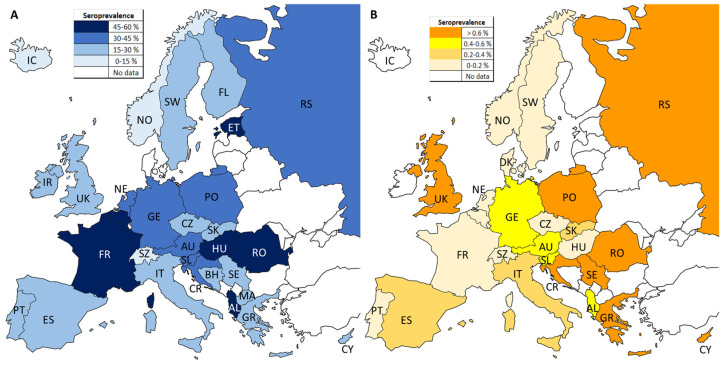
Pooled seroprevalences of *T. gondii* IgG (**A**) and IgM (**B**) antibodies in European residents between 2000 and 2020. AL (Albania), AU (Austria), BH (Bosnia and Herzegovina), CR (Croatia), CY (Cyprus), CZ (Czech Republic), ES (Spain), ET (Estonia), FL (Finland), GE (Germany), GR (Greece), HU (Hungary), IC (Iceland), IR (Ireland), IT (Italy), MA (North Macedonia), NE (Netherlands), NO (Norway), PO (Poland), PT (Portugal), RO (Romania), RS (Russia), SE (Serbia), SK (Slovakia), SL (Slovenia), SW (Sweden), SZ (Switzerland), UK (United Kingdom).

Figure 2A illustrates the pooled *T. gondii* IgG seroprevalence values by European country. The overall IgG seroprevalence was 32.1% (95% CI 29.0–35.2), with values of 20.1% (95% CI 16.8–23.5), 38.5% (95% CI 29.7–47.2), 39.7% (95% CI 32.1–47.2), and 27.5% (95% CI 24.3–30.7) for North, West, East, and South regions, respectively, and statistically significant differences (Student’s *t*-test) were observed between areas (*p* < 0.05) except between the West and East areas (*p* < 0.8384). 

Regarding target populations, the IgG seroprevalences were 38.6% (95% CI 33.7–43.6) for the general population (e.g., healthy people of different ages), 28.3% (95% CI 24.2–32.4) for pregnant women or women of childbearing age, and 31.1% (95% CI 29.0–33.1) for other groups of the population considered to be people at higher risk (e.g., health care workers, abattoir workers, farmers, hospital patients, etc.). Statistically significant differences were observed for seroprevalence in the general population compared to that of pregnant women/women of childbearing age (*p* = 0.011). 

The frequencies obtained of *T. gondii* IgG antibodies for the type of diagnostic method used were 30.2% (95% CI 28.1–32.3), 40.1% (95% CI 35.4–44.8), and 43.2% (95% CI 35.4–51.0) for commercial, in-house, and a combination of both, respectively, and a statistically significant difference (*p* = 0.011) was observed when commercial and in-house methods were compared. Supplementary Figures S1 and S2 present the forest plot of the seroprevalence of IgG antibodies by country and funnel plots of IgG seroprevalence by the variables analyzed (region, type of population, and analytical methods), respectively. A high heterogeneity (I^2^ = 100%, *p* < 0.001; Q = 3.5 × 10^5^ (d.f. = 135), *p* < 0.001) and degree of publication bias (Egger’s test = 6.14, *p* < 0.001) were observed among the 134 studies considered.

### 3.2. Seroprevalence of Anti-T. gondii IgM in European Residents

Figure 2B illustrates the anti-*T. gondii* IgM results per European country, and Table 3 summarizes the characteristics of the 88 eligible studies. The pooled *T. gondii* IgM seroprevalence was 0.6% (95% CI 0.5–0.6), with values of 0.1% (95% CI 0.0–0.1), 0.2% (95% CI 0.1–0.3), 1.5% (95% CI 1.3–1.8), and 1.1% (95% CI 1.0–1.3) for the North, West, East, and South regions, respectively. Statistically significant differences (Student’s *t*-test) were observed between all areas (*p* < 0.001) except between the North and West (*p* = 0.073). A high heterogeneity (I^2^ = 99.1%, *p* < 0.001; Q = 6697.26 (d.f. = 66), *p* < 0.001) and degree of publication bias (Egger’s test = 6.52, *p* < 0.001) were observed among the 67 studies considered for statistical analyses.

Most of the studies (65.9%; 58/88) focused on the detection of IgM antibodies in pregnant women or newborn children as the main target populations; as expected, different frequencies of IgM detection were observed: 0.1% (95% CI 0.0–0.1) in newborns (8 studies), 0.9% (95% CI 0.8–1.0) in pregnant women/childbearing-age women (47 studies), 1.6% (95% CI 1.2–2.0) in the general population (24 studies), and 2.5% (95% CI 1.0–4.0) in other groups (e.g., sick people) (6 studies). Again, the population-type subgroup analysis demonstrated a high heterogeneity (I^2^ = 98.8%, *p* < 0.001; Q = 7577.96 (d.f. = 88), *p* < 0.001) and degree of publication bias (Egger’s test = 26.02, *p* < 0.001) among the 88 studies considered. 

### 3.3. Identification of Risk Factors

**Figure 3:**
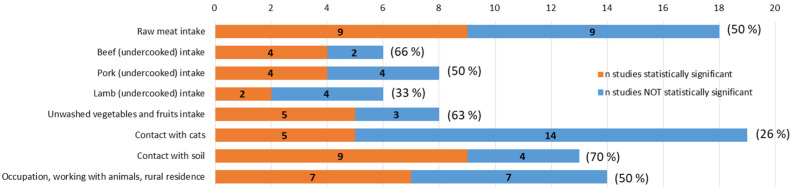
Summary of the risk factors identified for *T. gondii* infection in human populations residing in Europe. Each bar indicates the number of studies that considered such potential risk factors and the numbers of them resulting in a statistical association were evaluated with odds ratio or relative risk. Brackets indicate the proportions of studies considering such factors as statistically significant (*p* < 0.05). Raw data are presented in Supplementary Table S2.

Thirty studies reported data from 23 countries on risk factors for *T. gondii* infection (Supplementary Table S2). Among those, one study was multicentric, involving five countries (Belgium, Denmark, Italy, Switzerland, and Norway) from the North, South, and West areas [11], and two studies were undertaken in two East countries (Czech Republic and Slovakia) [168], and in three North countries (Sweden, Estonia, and Iceland) [23]. Among such heterogeneous studies, eight main risk factors were reported in Europe (Figure 3); within those, the most frequently investigated were “contact with cats” (63% [19/30] of studies), “consumption of raw (or undercooked) meat” without specification of the animal species of origin (60% [18/30]), “specific occupation of risk and working with animals” (47% [14/30]), and “contact with soil” (43% [13/30]); nevertheless, within each risk factor category and attending to the frequency/proportion of studies presenting associated statistical significance, “contact with soil” (9/13 [70%] studies), “intake of undercooked beef” (4/6 [66%] studies), and “intake of unwashed vegetables and fruits” (5/8 [63%] studies) stood out as demonstrated main facts associated with seroconversion and infections in European residents.

## 4. Discussion

The present study aimed at summarizing the anti-*Toxoplasma* IgG and IgM seroprevalence data of human populations residing in Europe contained in studies published between 2000 and 2020. Previous reviews [169,170,171,172] accomplished this task from a global point of view. In general, remarkable differences in the seroprevalence against *T. gondii* in different areas and continents were reported, in agreement with the fact that major routes of transmission differ in distinct human contexts involving different cultures, socioeconomic development, and culinary habits. 

The present review evidences a pooled overall seroprevalence in European residents (IgG: 32.1%; IgM: 0.6%) that is in the line with other international studies and areas of similar socioeconomic status (and habits). Comparable studies in the general population of the USA between 2011 and 2014 estimated that seroprevalence reached a low level of 11.14% (95% CI: 9.88–12.51) for IgG antibodies [173]. Indeed, an overall IgG seroprevalence of 17.5% in USA/Canada has been reported (reviewed in [171]). 

Despite being beyond the scope of the present review, a more specific systematic review [174] focused on the global prevalence of latent toxoplasmosis in pregnant women. The authors reported 31.2% seropositivity in European pregnant women (from 1988 to 2019), which is in agreement with data reported here (e.g., 28.3% in the present review). Nevertheless, current occurrences (e.g., last 5 years) may be lower, since the epidemiological situation has clearly evolved with the evidence provided by several long-term observational studies. Two of these deserve attention: a French survey focused on pregnant women reported a decrease in IgG prevalence from 55.0% in 1995 to 33.7% in 2016 [175], and another comprehensive investigation from Austria reported a yearly decline in IgG seroprevalence of 0.56% in the time period 1995–2006, and a 1.20% annual decline from 2006 to 2012; in sum, IgG seroprevalence decreased from 43.3% in 1995 to 31.5% in 2012 [43]. Both countries have a well-established national surveillance system [176]. As a consequence, national (local/regional) longitudinal studies in different population strata (e.g., health workers, children, etc.) are clearly necessary to assess the true expected decreasing trends in the remaining European countries.

One limitation of the present review is that the mean age of female participants from each study was unknown. This demographic nuance may create the illusion of deflated prevalence rates among pregnant women in specific areas. Therefore, readjustments of the prevalence to standard ages through mathematical procedures would have been desirable to overcome such a bias [177,178].

As usually reported, the presence of IgM is investigated as an indicator of recent *Toxoplasma* infection, which is the rationale for the majority (66%) of surveys targeting pregnant women or newborn children (Table 3). The seroprevalence values observed in European regions (range: 0.1–1.1%) are in accord with other areas of similar sociodemographic level, such as the USA (1.16% (95% CI: 0.94–1.42) [173]. Despite IgM figures being of epidemiological interest, additional extra effort should be made in many countries to estimate the incidence of clinical toxoplasmosis and its further report (e.g., congenital toxoplasmosis) [8].

Detailed and harmonized data compilation results are of great interest for comparisons among areas, but especially when comprehensive data analyses (e.g., association by logistic regression models) are desired [2,179,180]. In this regard, the lack of harmonization especially among the available serological diagnostic methods [181] can hinder the possibilities of making comparisons between studies; this is a fact that is frequently observed in other meta-analyses, in which the heterogeneity values observed used to be high (>75%) [20,174].

Despite the remarkable degree of heterogeneity observed, the frequency of exposure to *T. gondii* is also high and widespread; therefore, unraveling the most common route of infection is highly desirable to design appropriate intervention strategies.

Currently, the relative importance of meat-borne vs. oocyst-driven transmission of *T. gondii* is little known. A review of the literature indicates that 30–60% of infections could be attributed to meat as the infection source, while 6–17% could be attributed to contact with soil or other environmental matrices containing oocysts [11,15,182]. Therefore, studies aiming to unravel what the main sources of infection are for European residents are of major interest [14,16]. 

Systematically compiling the information is key for the identification of patterns related to toxoplasmosis outbreaks [15,172,183]. Whether the severity of clinical infections in humans is associated with one of such routes is not known; however, in laboratory animals, oocysts, as a source of infection, cause more severe clinical disease [172]. A worldwide systematic review of *T. gondii* outbreaks [183] selected 38 studies reporting details regarding epidemiological data and dynamic of infections. Some findings deserve attention, notably, a large number of individuals were affected when oocysts were the suspected or confirmed source of infection, and a broader and prolonged appearance of new cases occurred via such a source when compared to tissue cysts. There is limited information regarding Europe, where very few outbreaks have been observed, which were associated with ingestion of unpasteurized milk [184] and undercooked meat [185,186,187].

More recently, additional data on the patterns of transmission and source of infection in global outbreaks of human toxoplasmosis have been reported [15]; authors suggested that transmission routes presented variations by decade. For example, in the 1960s and 1990s, ingestion of cysts in meat and meat products were considered the main sources of infection; in the 1980s, milk contaminated with tachyzoites; in the 2000s, the outbreaks were more related to the presence of oocysts in water, sand, and soil; and after 2010, due to oocysts in raw fruits and vegetables. Therefore, additional studies on the source attribution that aim to identify the true source of infection are warranted [17]. 

Despite very few toxoplasmosis outbreaks having been reported in Europe [183], exposure to the parasite is frequent, and major risk factors have been identified based on its statistical association with infections. Knowledge of risk factors related to diet, hygiene practices, and lifestyle will help to target prevention efforts. In the present review (Supplementary Table S2), the eight worldwide main risk factors related to the meat-borne and the environmental routes were considered [14]. Among the 30 studies reporting such information in the last 20 years in Europe, three risk factors (“contact with soil”, “consumption of undercooked beef”, and “intake of unwashed vegetables”) stood out as the most frequently associated to seroconversion in people. 

Considering those related to the meat-borne route, 66% of studies identified the “intake of undercooked beef” as a risk factor; despite cattle livestock being considered a less susceptible host for *T. gondii* [1], its meat, unlike others, is frequently (and traditionally) consumed undercooked in Europe. This risk factor had been identified in a worldwide review [12] based on case–control studies aimed at assessing the risk of humans developing acute *T. gondii* infections; indeed, findings related to the consumption of raw/undercooked beef (OR = 2.22; 95% CI: 1.57–3.12), raw/undercooked sheep meat (OR = 3.85; 95% CI: 1.85–8.00), and unspecified meat (consumption of raw/undercooked meat (OR = 3.44; 95% CI: 1.29–9.16) demonstrated the importance of the meat-borne route. Previous studies carried out prior to the 2000s in Europe identified the “consumption of undercooked beef” (OR = 5.5; 95% CI: 1.1–27) and “consumption of undercooked lamb” (OR = 3.1; 95% CI: 0.85–14) as potential risk factors for primary infections during pregnancy in France [188]; by contrast, for the same segment of the population in Naples (Italy), “consumption of cured pork” (OR = 2.9; 95% CI: 1.6–5.5) and “raw meat” (OR = 2.6; 95% CI: 1.4–4.7) were the most significant elements [189]. In the same target population, “eating raw or undercooked minced meat products” (OR = 4.1; 95% CI: 1.5–11.2), “eating raw or undercooked mutton” (OR = 11.4; 95% CI: 2.1–63.1), and “eating raw or undercooked pork” (OR = 3.4; 95% CI: 1.1–10.4), were recognized in Norway [190]. Finally, in the USA, an area of similar socioeconomic development like Europe, very close factors like “eating raw ground beef” (OR = 6.67; 95% CI: 2.09–21.24), “eating rare lamb” (OR = 8.39; 95% CI: 3.68–19.16), and “eating locally produced cured, dried, or smoked meat” (OR = 1.97; 95% CI: 1.18–3.28) were identified through a case–control study [191].

Nevertheless, in the present review, consumption of other products of animal origin (raw eggs, and unpasteurized milk) was proven to be a non-significant risk factor, unlike in other areas such as the USA where drinking unpasteurized goat’s milk was a risk factor (OR = 5.09; 95% CI: 1.45–17.80) [191] and has been recognized as a cause of outbreaks [183].

Among the risk factors related to the environmental route [192], “contact with soil” and “intake of unwashed vegetables and fruits” were the most frequently identified (70% and 63% of studies, respectively) in the present review; both are related to *T. gondii* oocysts contamination [16] that is the product of sexual multiplication of the parasite in the gut of felines, which is quite resistant to environmental harmful conditions; a single sporulated oocyst is capable of producing the infection [1]. In European studies prior to 2000, “frequent consumption of raw vegetables outside the home” (OR = 3.1; 95% CI: 1.2–7.7) [188] and “eating unwashed raw vegetables or fruits” (OR = 2.4; 95% CI: 1.1–5.6) [190] had also been identified as risk factors. Also, in the USA [191], “eating raw oysters, clams, or mussels” (OR = 2.22; 95% CI, 1.07–4.61; AR, 16%) was significant in a separate model among persons asked this question, reinforcing the importance of environmental routes for human infections as demonstrated during outbreaks investigations [15]. In this regard, the remarkable frequency of detection of *T. gondii* oocysts in fresh produce, mollusk bivalves, and water bodies worldwide [16] increases research interest to specifically investigate the relative importance of the environmental route vs. the meat-borne route.

Among other recognized risk factors, “contact with cats” was associated with *T. gondii* infection in 5 of the 19 studies (Supplementary Table S2), where the variable was considered despite the well-established low frequency of cats shedding oocysts at a given time [22]. Such a risk factor had not been considered in previous studies carried out in Europe [189]; however, in the USA, “having three or more kittens” was significantly associated with infections (OR = 27.89; 95% CI: 5.72–135.86) [191]. Despite the epidemiological importance of cats in *T. gondii* transmission, contact with cats was also not the predominant risk factor for infection in studies carried out in other parts of the world [12,14]; it was not even identified in the first European multicenter study focused on pregnant women residing in six cities of five countries (Belgium, Denmark, Italy, Norway, and Switzerland) [11]. Other risk factors like “gardening” and “traveling out of Europe or the USA” have been recognized as significant [189,190,191]. Finally, the same authors [173] proposed some sociodemographic risk factors via a full logistic regression model (e.g., ethnic origin, place of birth, crowding, etc.); similar studies have been carried out for European populations (e.g., [154]).

As observed in the present review (Supplementary Table S2) for Europe, and in agreement with studies carried out in other geographical scenarios, most investigations focused on recent/primary infections in pregnant women. In a recent review [14] considering 187 case–control, cohort, and cross-sectional studies conducted worldwide between 1983 and 2016, the authors showed a long list of potential risk factors and highlighted the association of *T. gondii* sporadic infections with a wide range of environmental factors, along with those related to food habits. The authors declared that the main limitation for making inferences from the data (data interpretation) were the broad definition of exposures and the use of serological methods for the case definition. In this very same context, a major limitation arises; most of the worldwide studies reported risk factors related to *T. gondii* infections without taking the moment of infection into account (e.g., based upon IgG seropositivity) [13]. Indeed, new studies are necessary to clarify the major sources of infections for the human population in the EU. In this regard, a recent paper in the Netherlands [13] identified the importance of hand hygiene, and the need for detailed enquiries (including risk assessment) with specific meat types. 

Despite the present review’s focus on risk factors related to both meat-borne and environmental route-derived infections, further studies also addressing neglected risk factors (e.g., injuries caused by animals, sexual contact, etc.) [168,193,194,195] would be of interest.

The interest of regional-specific analyses to identify specific risk factors (e.g., case–control studies with country-specific products [13]) and the implementation of tools like quantitative microbial risk assessment (QMRA) methods [196] will clarify the current epidemiological scenario. Therefore, an integrative analysis from a One Health point of view will be of major interest to unravel and quantify the sources of *T. gondii* infections for human populations in Europe, which, by contrast, are subject to rapid change.

In summary, several needs are identified: source attributions and updated risk factors studies are missing in most countries under situations of rapid changes in food preferences and trends. A brief summary of each of the elements is presented below.

(a) There is a lack of seroprevalence data from several countries (e.g., Baltic states).
(b) Apparent regional differences may be due to the absence of harmonized data (e.g., type of target population), but also due to differences in a country’s culinary customs and sociodemographic development index.
(c) The high heterogeneity observed indicates the lack of harmonization of approaches (e.g., diagnostic methods) for *T. gondii* seroprevalence investigations in Europe. Given the high heterogeneity observed in the present study, it is clear that surveillance systems should first be implemented, and then harmonized in European countries [6,7,176].
(d) Development of up-to-date risk assessment (local/regional) studies taking into consideration particular trends are needed.

The overall results show that a noticeable segment of the European population (one-third) harbors anti-*Toxoplasma* IgG antibodies. Therefore, exposure to *T. gondii* seems to be frequent, and should be considered especially among people at risk. However, under the view of scattered and fragmented available data, a harmonized system for *Toxoplasma* infection surveillance in European countries is missing. In addition, extra efforts should be made to implement updated risk-factor analyses focusing on detailed source attribution in such a dynamic and evolving society, since culinary customs (and preferences) are rapidly changing.

## 5. Conclusions

Therefore, exposure to *T. gondii* seems to be frequent among European residents, and should be considered especially among vulnerable people. However, under the view of the scattered and fragmented data available, a harmonized system for *Toxoplasma* infection surveillance should be implemented in Europe, in order to better estimate the epidemiological scenario regarding *T. gondii* infection and its potential clinical toxoplasmosis (e.g., congenital toxoplasmosis). In addition, extra effort should be made to implement updated local risk-factor analyses focusing on detailed source attribution in such a dynamic and evolving society, since culinary customs (and preferences) are rapidly changing.

## Figures and Tables

**Table 1:** Summary of selected studies on seroprevalence of anti-*T. gondii* antibodies (IgG) in European residents published between 2000 and 2020.

Region/Country	Year	Population	Serological Method *	Commercial/In-House	Samples		Reference
					Total (*n*)	Positive (%)	
**North**							
Estonia	1999–2001	General population	ELISA	CM	215	118 (54.88)	[23]
	2004–2011	General population	ELISA	CM	999	557 (55.75)	[24]
	2004–2011	Other	ELISA	CM	925	539 (58.27)	
Finland	2000–2001	General population	MEIA, ELISA	CM	6250	1231 (19.69)	[25,26]
	2009	Other	ELFA	CM	294	43 (14.62)	[27]
Iceland	1999–2001	General population	ELISA	CM	440	43 (9.77)	[23,28]
Ireland	NR	Pregnant women	MAT	In-house	20,252	4991 (24.64)	[29]
Norway	1992–1994	Pregnant women	ELISA	CM	29,912	2937 (9.81)	[30]
	1994–2005	Other	ELISA	CM	1073	124 (11.55)	[31]
	2000	Pregnant women	NR	NR	361	43 (11.91)	[32]
	2003	Other	ELISA	CM	620	50 (8.06)	[33]
	2009	Pregnant women	MEIA	CM	206	35 (16.99)	[34]
	2010–2011	Pregnant women	ELISA	CM	1922	179 (9.31)	[35]
Sweden	1997–1998	Pregnant women	MEIA	CM	40,978	7390 (18.03)	[36,37]
	1999–2001	General population	ELISA	CM	361	83 (22.99)	[23]
United Kingdom	1999	Other	DAT	CM	425	191 (44.94)	[38]
	1999–2001	Pregnant women	MEIA	CM	1897	172 (9.06)	[39]
	2006–2008	Pregnant women	ELISA	CM	2610	452 (17.31)	[40]
	2006–2009	Other	ELISA	In-house	1403	185 (13.18)	[41]
	2012–2015	General population	ELFA	CM	5787	930 (16.07)	[42]
**West**							
Austria	1995–2012	Pregnant women	Several	In-house, CM	10,3316	3864 (37.39)	[43]
	2000	Other	IFAT	In-house	60	32 (53.33)	[44]
	2000–2007	Pregnant women	IFAT	In-house, CM	63,416	20,103 (31.70)	[45]
	2001–2002	Pregnant women	SF	In-house	5545	1830 (33.00)	[46]
Belgium	1991–2001	Pregnant women	NR	NR	16,541	8049 (48.66)	[47]
France	1995	Pregnant women	NR	NR	10,839	6806 (62.79)	[48]
	1995, 2003, 2010	Other	NR	NR	42,886	19,015 (44.33)	[49]
	1997–2013	General population	ELISA	CM	21,480	12,914 (60.12)	[50]
	2000	Other	NR	NR	262	50 (19.08)	[51]
	2004	Other	ELISA	CM	273	128 (46.88)	[52]
	2008–2009	General population	Several	CM	2060	1141 (55.38)	[53]
Germany	2008–2011	General population	ELFA	CM	6564	3602 (54.87)	[54]
	2008–2011	Pregnant women	ELISA	CM	5402	1856 (34.35)	[55]
Netherlands	1995–1996	General population	ELISA	In-house	7521	3046 (40.49)	[56]
	2006–2007	General population	ELISA	In-house	5541	1441 (26.00)	[57]
Switzerland	1982–1999	Other	IFAT	CM	64,622	1806 (2.79)	[58]
	2000–2010	Other	ELISA	CM	54,216	749 (1.38)	[59]
**East**							
Czech Republic	1988–2006	Other	Several	In-house, CM	626	268 (42.81)	[60]
	1988–2012	Other	MEIA	CM	1130	474 (41.94)	[61]
	2000–2004	Other	Several	CM	3250	757 (23.29)	[62]
	NR	General population	ELISA	CM	290	93 (32.06)	[63]
	NR	Pregnant women	NR	NR	144	23 (15.97)	[64]
		Other	NR	NR	144	23 (15.97)	
Hungary	1987–2000	Pregnant women	Several	In-house, CM	31,759	18,420 (55.99)	[65]
Poland	1991–2000	General population	Several	In-house, CM	9661	5297 (54.82)	[66]
	1996–1999	Other	MEIA	CM	985	532 (54.01)	[67]
	1998	Pregnant women	MEIA	CM	1920	837 (43.59)	[68]
	1998–2000	Other	DAT	CM	2684	19 (0.70)	[69]
	1998–2003	Pregnant women	Several	CM	4916	2030 (41.29)	[70]
	2000	Pregnant women	MEIA	CM	2016	722 (35.81)	[71]
	2000–2003	General population	MEIA	CM	4682	2574 (54.97)	[72]
	2003–2005	Other	ELISA	CM	784	490 (62.50)	[73]
	2004–2012	Pregnant women	ELFA	CM	8281	3364 (40.62)	[74]
	2005	General population	Several	In-house, CM	991	590 (59.53)	[75]
	2007–2010	Pregnant women	MEIA	CM	55	28 (50.90)	[76]
	2008	Other	ELFA	CM	537	292 (54.37)	[77]
	2010–2012	Other	IFAT	In-house	169	93 (55.02)	[78]
	2013–2014	General population	NR	NR	664	445 (67.01)	[79]
	2013–2014	Other	NR	NR	74	37 (50.00)	
	2014–2015	Other	DAT	CM	148	57 (38.51)	[80]
	2015–2016	Other	NR	NR	537	103 (19.18)	[81]
	2016–2017	Pregnant women	NR	NR	628	124 (19.74)	[82]
	2017	Other	ELFA	CM	373	166 (44.50)	[83]
	NR	Other	DAT	In-house	1497	864 (57.71)	[84]
	NR	General population	DAT	In-house	61	34 (55.73)	[85]
	NR	Other	DAT	In-house	107	70 (65.42)	
	NR	General population	ELFA	CM	293	186 (63.48)	[86]
	NR	Other	IFAT	In-house	190	77 (40.52)	[87]
Romania	2001–2006	Pregnant women	ELISA	CM	248	92 (37.09)	[88]
	2005–2007	Pregnant women	ELISA	CM	510	198 (38.82)	[89]
	2007	General population	Several	CM	1155	687 (59.48)	[90]
	2011	General population	DAT	CM	304	197 (64.8)	[91]
	2013	Other	ELISA	CM	51	24 (47.05)	[92]
	2018	Other	LAT	CM	441	73 (16.55)	[93]
	NR	Other	DAT	CM	184	106 (57.60)	[94]
Russia	1996–2002	Pregnant women	NR	NR	87,099	45,373 (52.09)	[95]
	1997–2006	General population	Several	CM	23,024	6250 (27.14)	[96]
	2006–2007	Other	NR	NR	4155	139 (3.34)	[97]
	2012	Other	CMIA	CM	77	4 (5.19)	[98]
	2013	General population	ELISA	CM	181	56 (30.93)	[99]
	2015	General population	ELISA	CM	1272	323 (25.39)	[100]
Slovakia	2000–2004	Pregnant women	ELISA	CM	656	145 (22.1)	[101]
	2003	General population	ELISA	CM	508	123 (24.21)	[102]
	2006	Other	ELISA	CM	118	40 (33.89)	[103]
	2007	General population	Several	In-house, CM	1845	577 (31.27)	[104]
	2011	General population	MEIA	CM	806	282 (34.98)	[105]
	NR	General population	MEIA	CM	1536	322 (20.96)	[106]
**South**							
Albania	2004–2005	Pregnant women	ELFA	CM	496	241 (48.58)	[107]
Bosnia and Herzegovina	2015	General population	DAT	In-house	320	98 (30.62)	[108]
Croatia	1994–1995	General population	ELISA	CM	1109	423 (38.14)	[109]
	1994–1996	General population	ELISA	CM	1464	533 (36.4)	[110]
	2000–2001	General population	MEIA	CM	219	115 (52.51)	[111]
	2000–2001	Other	MEIA	CM	166	86 (51.80)	
	2005–2009	Other	ELFA	CM	502	146 (29.08)	[112]
Cyprus	2009–2011	Other	ELISA	CM	1056	69 (6.53)	[113]
	2009–2014	Pregnant women	CMIA	CM	23,076	4129 (17.89)	
Greece	1984, 1994, 2004	General population	Several	In-house, CM	2784	851 (30.56)	[114]
	1985, 1998	Other	ELISA	CM	469	124 (26.43)	[115]
	1998–2003	Pregnant women	ELISA	CM	5532	1628 (29.42)	[116]
	1998–2005	Pregnant women	ELISA	CM	12,000	3540 (29.50)	[117]
Italy	1996–2000	Pregnant women	ELISA	CM	8061	2773 (34.40)	[118]
	1996–2000	Other	ELISA	CM	9730	5 (0.05)	
	2001–2012	Pregnant women	CMIA	CM	10,232	2814 (27.50)	[119]
	2004–2005	Pregnant women	ELISA	CM	3426	737 (21.51)	[120]
	2005–2006	Pregnant women	ELFA	CM	1501	281 (18.72)	[121]
	2005–2006	Pregnant women	ELFA	CM	892	319 (35.76)	[122]
	2005–2007	Pregnant women	ELISA	CM	2356	564 (23.93)	[123]
	2007–2009	General population	MEIA	CM	10,352	2216 (21.40)	[124]
	2007–2010	General population	MEIA	CM	13,177	3626 (27.51)	[125]
	2007–2014	Pregnant women	NR	NR	38,712	9368 (24.19)	[126]
	2009–2018	Pregnant women	CMIA	CM	45,492	9792 (21.52)	[127]
	2009–2011	Pregnant women	MEIA	CM	10,347	2308 (22.30)	[128]
	2010–2013	General population	ELFA	CM	12,306	3476 (28.24)	[129]
	2011–2015	Other	ELFA	CM	339	89 (26.25)	[130]
	2012	Pregnant women	NR	NR	846	152 (17.96)	[131]
	2013–2017	Other	ELISA	CM	1020	169 (16.56)	[132]
North Macedonia	2004–2005	Pregnant women	MEIA	CM	235	48 (20.42)	[133]
Portugal	2000–2015	Pregnant women	NR	NR	4060	1055 (25.98)	[134]
	2001–2002, 2013	General population	Several	CM	3097	913 (29.48)	[135]
	2004–2009	Pregnant women	NR	NR	3126	804 (25.71)	[136]
	2009–2010	Other	CMIA	CM	401	98 (24.43)	[137]
	2010–2011	Pregnant women	DAT	CM	155	34 (21.93)	[138]
Serbia	2001–2005	Other	DAT	In-house	765	249 (32.54)	[139]
	2005	Pregnant women	MEIA	CM	334	97 (29.04)	[140]
	2011–2012	Other	ELISA	CM	79	19 (24.05)	[141]
	NR	Pregnant women	ELISA	CM	662	180 (27.19)	[142]
Slovenia	1995–2002	Other	IFAT	In-house	413	236 (57.14)	[143]
	1996–1999	Pregnant women	IFAT	In-house	21,270	7151 (33.62)	[144]
Spain	1987–2001	Pregnant women	MEIA	CM	2796	19 (0.67)	[145]
	1992–1999	Other	ELFA	CM	7090	3009 (42.44)	[146]
	1992–2008	Pregnant women	MEIA	CM	47,635	15,196 (31.90)	[147]
	1999–2000	Other	Several	CM	157	56 (35.66)	[148]
	1999	Pregnant women	NR	NR	16,362	4687 (28.64)	[149]
	2001	Pregnant women	MEIA	CM	2929	552 (18.84)	[150]
	2002–2003	General population	MEIA	CM	2660	935 (35.15)	[151]
	2006	Pregnant women	ELISA	CM	699	183 (26.18)	[152]
	2006	Pregnant women	MEIA	CM	2623	550 (20.96)	[153]
	2006–2010	Pregnant women	ELISA	CM	2933	798 (27.20)	[154]
	2007–2008	Pregnant women	ELISA	CM	3541	602 (17.00)	[155]
	2007–2008	Pregnant women	MEIA	CM	1427	433 (30.34)	[156]
	2007–2010	Pregnant women	ELISA	CM	8012	1874 (23.38)	[157]

NR: not reported; CM: commercial system/kit; CMIA: chemiluminescent microparticle immunoassay; DAT: direct agglutination test; ELISA: enzyme-linked immunosorbent assay; ELFA: enzyme-linked fluorescence assay; IFAT: immunofluorescent antibody test; MEIA: microparticle enzyme immunoassay; SF: Sabin–Feldman dye test. * Studies included here in which analytical methods were not reported (NR) were selected because of the interest of the whole publication in the specific geographical context (e.g., representativeness).

**Table 2:** Subgroup analysis for comparison of seroprevalence of anti-*T. gondii* antibodies (IgG) in European residents published between 2000 and 2020.

Factor	No. of Studies Included	Pooled Seroprevalence (95% CI)	Heterogeneity Test				Egger’s Test	
			I^2^ (%)	Q (X^2^)	Q/df	Q-p (*p)*	*t*	*p*
**Area**								
North	19	20.1 (16.8–23.5)	99.6	4261.17	19	<0.001	1.14	0.267
West	17	38.5 (29.7–47.2)	100.0	1.7 × 10^5^	17	<0.001	4.11	0.001
East	47	39.7 (32.1–47.2)	99.9	77,136.83	46	<0.001	1.18	0.243
South	51	27.5 (24.4–30.7)	99.8	27,079.87	50	<0.001	2.71	0.009
**Population type**								
General	38	38.6 (33.7–43.6)	99.8	17,521.25	37	<0.001	0.79	0.432
Pregnant women	58	28.3 (24.2–32.4)	99.9	98,992.78	57	<0.001	0.50	0.619
Other	47	31.1 (29.0–33.1)	99.9	53,155.52	46	<0.001	4.70	<0.001
**Diagnostic method**								
Commercial	101	30.2 (28.1–32.3)	99.9	1.8 × 10^5^	100	<0.001	9.66	<0.001
In-house	15	40.1 (35.4–44.8)	99.3	1930.18	14	<0.001	1.49	0.161
Both	8	43.2 (35.4–51.0)	99.9	7675.92	7	<0.001	0.71	0.507
**Overall**	134	32.1 (29.0–35.2)	100.0	3.5 × 10^5^	135	<0.001	6.14	<0.001

I^2^, inverse variance index; Q, Cochran’s X^2^; Q-p, *p*-value of Q-tests.

**Table 3:** Summary of studies reporting anti-*T. gondii* antibodies (IgM) in European residents published between 2000 and 2020.

Region/Country	Year	Population	Serological Method *	Commercial/In-House	Samples		Reference
					Total (*n*)	Positive (%)	
**North**							
Denmark	1999–2002	Newborns	ISAGA	CM	262,912	96 (0.04)	[158]
	1999–2007	Newborns	ISAGA	CM	547,820	100 (0.02)	[159]
Norway	1992–1994	Pregnant women	ELISA	CM	35,940	47 (0.13)	[30]
Sweden	1997–1998	Newborns	ISAGA, ELISA	CM	40,978	45 (0.11)	[160]
	1997–1998	Newborns	MEIA	CM	40,978	3 (0.01)	[37]
United Kingdom	1999–2001	Pregnant women	MEIA	CM	1897	12 (0.63)	[39]
**West**							
Austria	1995–2012	Pregnant women	ELFA, MEIA	CM	103,316	878 (0.85)	[43]
	2000–2005	Pregnant women	ELFA	CM	51,754	51 (0.10)	[161]
	2000–2007	Pregnant women	ELFA	CM	63,416	66 (0.10)	[45]
	2001–2002	Parturient women	NR	-	5545	7 (0.13)	[46]
Belgium	1991–2001	Pregnant women	NR	-	16,541	8 (0.05)	[47]
France	2004	General population	ELISA	CM	273	0 (0.00)	[52]
Germany	2008–2011	Pregnant women	ELISA	CM	4011	17 (0.42)	[55]
Netherlands	2006	Newborns	ISAGA	CM	10,008	18 (0.18)	[162]
Switzerland	1986–1999	Women giving birth	ELFA	CM	64,622	107 (0.16)	[58]
	2000–2015	Women giving birth	ELISA, MEIA	CM	54,216	51 (0.09)	[59]
**East**							
Czech Republic	1988–2006	Other (HIV+ patients)	ELISA	CM	626	5 (8.06)	[60]
	2000–2004	Other	CFT, ELISA	CM	3250	1 (0.03)	[62]
Hungary	1987–2000	Pregnant women	ELISA	CM	31,759	20 (0.06)	[65]
Poland	1996–1998	Newborns	ELFA, ELISA	CM	27,516	13 (0.05)	[163]
	1996–1999	Women of childbearing age	MEIA	CM	985	9 (0.91)	[67]
	1998–2000	Newborns	ELISA	CM	2684	15 (0.56)	[69]
	1998	Pregnant women	MEIA	CM	1920	27 (1.41)	[68]
	1998–2003	Pregnant women	ELISA	CM	4916	244 (4.96)	[70]
	1999–2000-2003	General population	MEIA	CM	4594	196 (4.27)	[72]
	2000	Pregnant women	ELFA	CM	2016	5 (0.25)	[71]
	2003–2005	General population	MEIA	CM	784	18 (2.29)	[73]
	2004–2012	Pregnant women	ELFA	CM	8281	803 (9.70)	[74]
	2007–2010	Pregnant women	MEIA	CM	55	18 (32.73)	[76]
	2008	General population	ELFA	CM	537	6 (1.12)	[77]
	2015–2016	Other (Transplant recipients)	NR	-	292	3 (1.03)	[81]
	2016–2017	Pregnant women	NR	-	628	2 (0.32)	[82]
	2017	Other (Veterinarians)	ELFA	CM	373	8 (2.14)	[83]
	NR	Other	ISAGA	CM	1497	3 (0.20)	[84]
	NR	Other	ELFA	CM	107	3 (2.80)	[85]
	NR	General population	ELFA	CM	61	0 (0.00)	[85]
	NR	General population	ELFA	CM	293	5 (1.71)	[86]
	NR	Children (8–16 yrs)	ELISA	CM	190	3 (1.57)	[87]
Romania	2001–2006	Pregnant women	ELISA	CM	248	4 (1.61)	[88]
	2005–2007	Pregnant women	ELISA	CM	510	46 (9.02)	[89]
	2007	General population	ELISA, MEIA	CM	1155	1 (0.09)	[90]
	2013	Other (Hematological malignancies)	ELISA	CM	51	0 (0.00)	[92]
Russia	1996–2002	Pregnant women	NR	-	87,099	1707 (1.96)	[95]
	2012	General population	MEIA	CM	77	0 (0.00)	[98]
Slovakia	2000–2004	Pregnant women	MEIA	CM	656	3 (0.46)	[101]
	2003	General population	MEIA	CM	508	0 (0.00)	[102]
	2007	General population	MEIA	CM	1845	9 (0.49)	[104]
**South**							
Albania	2004–2005	Pregnant women	ELFA	CM	496	3 (0.60)	[107]
Croatia	2000–2001	General population	MEIA	CM	219	2 (0.91)	[111]
	2000–2001	Other (HIV+ patients)	MEIA	CM	166	2 (1.20)	[111]
	2005–2009	Women of childbearing age	ELFA	CM	502	12 (2,39)	[112]
Cyprus	2008–2011	High school females (16–18 yrs)	ELISA	CM	1056	10 (0.95)	[113]
	2009–2011	Pregnant women	ELISA	CM	17,631	107 (0.60)	
Greece	1984–2004	General population	MEIA	CM	2784	42 (1.51)	[114]
	1998–2003	Pregnant women	ELISA	CM	5532	185 (3.34)	[116]
	1998–2005	Pregnant women	ELISA	CM	12,000	396 (3.30)	[117]
Italy	1996–2000	Pregnant women	MEIA	CM	8061	188 (2.33)	[118]
	2001–2012	Pregnant women	CMIA	CM	10,085	9 (0.09)	[119]
	2001–2012	Newborns	NR	-	738,588	1159 (0.16)	[164]
	2004–2005	Pregnant women	ELFA, ELISA	CM	3426	31 (0.90)	[120]
	2005–2006	Pregnant women	ELFA	CM	1501	23 (1.53)	[121]
	2005–2007	Pregnant women	ELISA	CM	2356	113 (4.80)	[123]
	2007–2009	General population	ELFA	CM	10,352	111 (1.07)	[124]
	2007–2010	General population	MEIA	CM	13,177	217 (1.65)	[125]
	2007–2014	Women giving birth	NR	-	38,712	110 (0.28)	[126]
	2009–2011	Pregnant women	ELISA	CM	10,347	80 (0.77)	[128]
	2009–2018	Women giving birth	CMIA	CM	45,492	123 (0.27)	[127]
	2010–2013	General population	ELFA	CM	12,306	164 (1.33)	[129]
	2011–2015	Pediatric patients	ISAGA, MEIA	CM	187	4 (2.14)	[130]
	2012	Pregnant women	NR	-	846	3 (0.35)	[131]
	2012–2014	Pregnant women	CMIA, ELFA	CM	36,877	156 (0.42)	[165]
	NR	Pregnant women	NR	-	892	7 (0.78)	[122]
Portugal	2000–2015	Newborns	NR	-	39,585	22 (0.05)	[134]
	2004–2009	Pregnant women	NR	-	3126	5 (0.16)	[136]
	2009–2010	Women of childbearing age	CMIA	CM	401	4 (1.00)	[137]
	2010–2011	Pregnant women	DAT	CM	155	17 (10.97)	[138]
Serbia	2001–2005	Women of childbearing age	ELISA, ISAGA	CM	765	53 (6.93)	[139]
	2004–2008	General population	ISAGA	CM	1106	77 (6.96)	[166]
	2005	Pregnant women	MEIA	CM	334	4 (1.20)	[140]
	2011–2012	Women of childbearing age	ELISA	CM	79	9 (11.39)	[141]
	NR	Pregnant women	ELISA	CM	662	13 (1.96)	[142]
Slovenia	1995–2002	Other (Ocular disease patients)	ELISA	CM	413	18 (4.36)	[143]
	1996–1999	Pregnant women	ELISA	CM	21,270	132 (0.62)	[144]
	1999–2004	Pregnant women	ELFA	CM	40,081	153 (0.38)	[167]
Spain	1987–2001	Pregnant women	ELISA, MEIA	CM	2796	19 (2.40)	[145]
	1992–2008	Pregnant women	ISAGA, MEIA	CM	47,635	24 (0.05)	[147]
	1999	Pregnant women	NR	-	16,362	106 (0.65)	[149]
	1999–2000	Other (HIV+ patients)	MEIA	CM	157	1 (1.75)	[148]
	2001	Pregnant women	MEIA	CM	2929	24 (0.82)	[150]
	2006	Pregnant women	MEIA	CM	2416	8 (0.33)	[153]
	2007–2008	Pregnant women	MEIA	CM	1427	12 (0.84)	[156]

NR: not reported; CM: commercial system/kit; CFT: complement fixation test; CMIA: chemiluminescent microparticle immunoassay; ISAGA: immunosorbent agglutination assay; ELFA: enzyme-linked fluorescence assay; ELISA: enzyme-linked immunosorbent assay; MEIA: microparticle enzyme immunoassay. * Studies included here in which analytical methods were not reported (NR) were selected because of the interest of the whole publication in the specific geographical context (e.g., representativeness).

## Data Availability

Not applicable.

## Supplementary Materials

The following supporting information can be downloaded at: https://www.mdpi.com/article/10.3390/pathogens12121430/s1, Table S1: PRISMA_2020 _checklist; Table S2: Main characteristics of the risk factor selected studies (*n* = 30) for *Toxoplasma gondii* infection in human residents in Europe. Supplementary Figure S1. Forest plot of the seroprevalence of anti-*Toxoplasma gondii* IgG antibodies by country. Figure has been constructed with IBM SPSS Statistics (Version 29.0, IBM Corp., Armonk, NY, USA). Supplementary Figure S2. Funnel plots of the seroprevalence of anti-*Toxoplasma gondii* IgG antibodies by each of the variables considered. Supplementary Figure S3. Forest plot of the seroprevalence of anti-*Toxoplasma gondii* IgM antibodies by country. References [197,198,199] were cited in the Supplementary Materials.
