# Supplementary material for: Anti-Toxoplasma gondii Antibodies in European Residents: A Systematic Review and Meta-Analysis of Studies Published between 2000 and 2020
Source: Pathogens. 2023 Dec 8;12(12):1430. doi: 10.3390/pathogens12121430 (PMC10745778; doi:10.3390/pathogens12121430)
Supplement: Supplementary file 1 [file pathogens-12-01430-s001.zip › Table S2 Summary data risk factors.pdf]

Supplementary Table S2: Main characteristics of the risk factor selected studies (n=30) for *Toxoplasma gondii* infection in human residents in Europe.

Study No.	Country (year)	No. of cases Population type	Consumption					Contact with cats	Contact with soil	Occupation working with animals/rural residence	Reference
			**Raw meat**	**Beef**	**Pork**	**Lamb**	**Unwashed vegetables and fruits**				
1	Europe (multicentric)* (1994-1995)	960	Taste raw meat while cooking OR: 4.7 (2.1-10.9); **p=0.001**	OR: 2.4 (1.6-3.4); **p=0.01**, undercooked	OR: 1.6 (0.8-3.0; p=0.18, undercooked	OR: 3.2 (1.5-6.9); **p=0.002**, undercooked	NR	OR: 1.0 (0.7-1.5); p=0.99	OR: 1.80 (1.1-2.9); **p=0.01**	OR: 1.9 (1.1-3.0) **p=0.01**	[11]
2	Bosnia and Herzegovina (2015)	320 blood donors	NR	NR	NR	NR	NR	NR	NR	Rural residence: OR: 1.83 (1.10-3.05); p=0.020	[108]
3	Cyprus (2008-2011)	854 high-school girls	NR	NR	Salami: OR:1.92 (1.10-3.34); **p=0.023**	NR	NR	OR: 2.51 (0.87-7.20); **p=0.05**	NR	NR	[113]
4	Czech and Slovak Republics (2014-2015)	1865	OR: 0.95 (0.64-1.41); p=0.79	NR	NR	NR	OR: 1.71 (1.02-2.87); **p=0.04**	Cat-related injuries OR: 2.16 (1.25-3.74); **p=0.01**<br><br>No. of cats at home OR: 1.14 (0.65-2.01); p=0.65	OR: 3.14 (1.3-6.35); **p<0.001**	NR	[168]

5	Czech Republic (2000-2004)	3250	OR: 1.39 (1.18-1.64); **p=0.001**	NR	NR	NR	NR	OR: 1.53 (1.30-1.81); **p=0.001**	NR	NR	[62]
6	Finland (2009)	294 veterinarians	OR: 0.73 (0.125-4.305); p=0.730	Raw beef while cooking: OR:3.36 (1.74-6.47); **p<0.001**	NR	Raw lamb while cooking: OR: 2.36 (0.94-5.89); p=0.0667	NR	NR	NR	Living in countryside OR: 4.003 (1.855-8.64); **p=0.001**	[27]
7	France (2004)	273	Meat but not raw OR: 1.33 (0.03-48.67); p=NR	OR: 1.08 (0.41-2.70); p=NR	OR: 0.81 (0.25-2.58); p=NR	OR: 2.13 (0.63-7.29); p=NR	OR: 5.88 (0.86-51.6); p=NR	OR: 1.31 (0.32-5.80); p=NR	Having garden OR: 0.53 (0.09-3.45); p=NR	OR: 1.89 (0.41-9.98); p=NR	[52]
8	Germany (NR)	6564	NR	NR	NR	NR	Vegetarian: OR: 0.56 (0.40-0.80) **p=0.048**	OR: 1.23 (0.97-1.32); p=0.127	NR	NR	[54]
9	Iceland (1999-2001)	442	NR	NR	NR	NR	NR	NR	NR	Rural residence: OR: 0.95 (0.79-4.87); p=NR	[28]
10	Italy (2005-2007)	113 congenital infections	OR: 6.53 (4.04-10.56); **p<0.001**	NR	Sausages: Marketed: OR: 4.18 (2.65-6.60); **p<0.001** Homemade: OR: 12.00 (6.74-21.38); **p<0.001**	NR	OR: 1.46 (0.93-2.27) p=0.0969	OR: 3.79 (2.36-6.08); **p<0.001**	Garden: OR: 2.76 (1.72-4.43); **p<0.001** Orchard: OR: 7.10 (3.17-15.91); **p<0.001**	Rural residence: OR: 13.80 (8.55-22.28); **p<0.001**	[123]

11	Macedonia (2004-2005)	235 pregnant women	**RR**: 2.43 (0.56-10.53); p=0.237	NR	NR	NR	NR	**RR**: 0.72 (0.28-1.84); p=0.491	**RR**: 1.946 (1.03-3.96); **p=0.042**	NR	[133]
12	Netherlands (2006-2007)	5541	Raw pork OR: 1.40 (1.23-1.59); **p<0.001**	NR	NR	NR	OR: 1.50 (1.0-2.1) p=NR	OR: 0.97 (0.86-1.10); p=0.682	NR	Farmers: OR: 1.25 (0.96-1.65); p=0.102	[57]
13	Poland (NR)	58 boys	Coefficient: 0.939, SE: 0.463, OR: 2.557, **p=0.0479**	NR	NR	NR	NR	Coefficient: 0.042, SE: 0.116, OR: 1.043, p=0.7163	NR	NR	[87]
		94 girls	Coefficient: 0.150, SE: 0.106, OR: 1.162, p=0.161	NR	NR	NR	NR	Coefficient: 0.067, SE: 0.389, OR: 1.069, p=0.864	NR	NR	
14	Poland (2013-2014)	78 childbearing age women	NR	NR	NR	NR	NR	NR	OR: 8.69 (2.93-25.76); **p<0.001**	Rural residence: OR: 2.77 (1.02-7.54); **p=0.0458**	[79]
15	Portugal (2009-2010)	401	OR: 1.5 (0.7-3.4); p=0.315	NR	OR: 2.5 (1.3-4.9); **p=0.009**	NR	OR: 7.6 (3.9-14.9); **p<0.001**	NR	OR: 8.4 (4.4-16.1); **p<0.001**	NR	[137]
16	Portugal (2010-2011)	155 pregnant women	NR	NR	NR	NR	NR	OR: 13.2 (1.43-121.90); **p=0.023**	NR	NR	[138]
17	Romania (2001-2006)	248 pregnant women	OR: 3.19 (0.386-26.43); p=0.285	NR	NR	NR	NR	NR	OR: 2.75 (0.330-22.89);	NR	[88]

									p=0.3495		
18	Romania (NR)	1155	NR	NR	NR	NR	NR	NR	NR	Rural residence: OR: 1.43 (1.11-1.83); **p=0.005**	[90]
19	Romania (2013)	51 haematological malignancies	NR	NR	NR	NR	NR	NR	NR	Contact with farm animals: OR: 11.1 (1.04-118.6); **p=0.05**	[92]
20	Romania (2018)	441 children	NR	NR	NR	NR	NR	NR	NR	Rural residence: OR: 1.31 (0.79-2.16); p=0.297	[93]
21	Serbia (1988-1997)	2936	Undercooked: **RR**: 1.6 (1.17-2.10); **p=0.009**	NR	NR	NR	NR	NR	**RR**: 10.3 (2.75-38.6); **p=0.022**	Suburban area: **RR**: 0.78 (0.75-0.81); **p=0.048**	[197]
22	Serbia (2001-2005)	765	**RR**: 1.84 (1.21-2.79); **p=0.008**	**RR**: 1.006 (1.001-1.011) **p=0.027**	NR, p=0.604	NR, p=0.496	NR	OR: 1.18 (0.88-1.57); p=0.259	**RR**: 1.68 (1.20-2.34); **p=0.002**	NR	[139]
23	Serbia (2004-2008)	69 clinical cases	OR: 7.67 (1.61-36.45); **p=0.010**	NR	NR	NR	NR	NR p=0.198	NR p=0.375	NR	[166]
24	Slovakia (2003)	508	OR: 0.60 (0.29-1.23); p=0.129	NR	NR	NR	OR: 0.6 (0.35-1.03); **p=0.049**	NR	OR: 1.21 (0.65-2.24); p=0.526	NR	[102]

25	Spain (1992-1999)	2883 childbearing age women	NR	NR	NR	NR	NR	NR	NR	Rural residence: OR: 1.18 (0.92-1.52); p=0.189	[146]
26	Spain (2002-2003)	2660	NR	NR	NR	NR	NR	OR: 1.22 (0.97-1.54); p=NR	NR	NR	[151]
27	Sweden, Estonia and Iceland (1999-2001)	1277	NR	NR	NR	NR	NR	Cat keeping in childhood OR: 1.20 (0.85-1.69); p=NR	NR	Rural residence: OR: 0.99 (0.68-1.42); p= NR	[23]
28	UK (1999-2001)	1897	NR	**RR**: 2.12 (0.88-5.1); p=0.346	**RR**: 9.56 (1.46-62.76); **p=0.043**	**RR**: 1.77 (0.20-15.33); p=0.624	NR	**RR**: 1.15 (0.86-1.54); p=0.393	Smoking while gardening: **RR**: 1.26 (0.82-1.94); **p=0.001**	NR	[39]
29	UK (2006-2007)	2610	OR: 1.64 (1.29-2.08); **p=0.001**	NR	NR	NR	To be vegetarian OR: 0.67 (0.45-0.99) **p=0.04**	OR: 0.79 (0.58-1.08); p=0.13	NR	NR	[40]
30	UK (England and Wales) (2012-2013)	55 cases of acute toxoplasmosis	OR: 0.74 (0.02-∞); p=0.28	OR: 10.7 (1.49-∞); **p<0.001**	OR: 2.70 (0.60-12.3); p=0.18	OR: 4.93 (1.29-18.8); **p=0.01**	NR	OR: 1.65 (0.46-5.91); p=0.44	OR: 1.83 (0.41-8.19); p=0.43	NR	[198]

*Involving centers from Belgium, Denmark, Italy, Switzerland and Norway; NR: data not reported; OR: odds ratio (case-control studies); RR: relative risk (retrospective cohorts studies); P-values are boldface when the comparison resulted statistically significant.
