# Supplementary figures and images for: Anti-Toxoplasma gondii Antibodies in European Residents: A Systematic Review and Meta-Analysis of Studies Published between 2000 and 2020
Source: Pathogens. 2023 Dec 8;12(12):1430. doi: 10.3390/pathogens12121430 (PMC10745778; doi:10.3390/pathogens12121430)

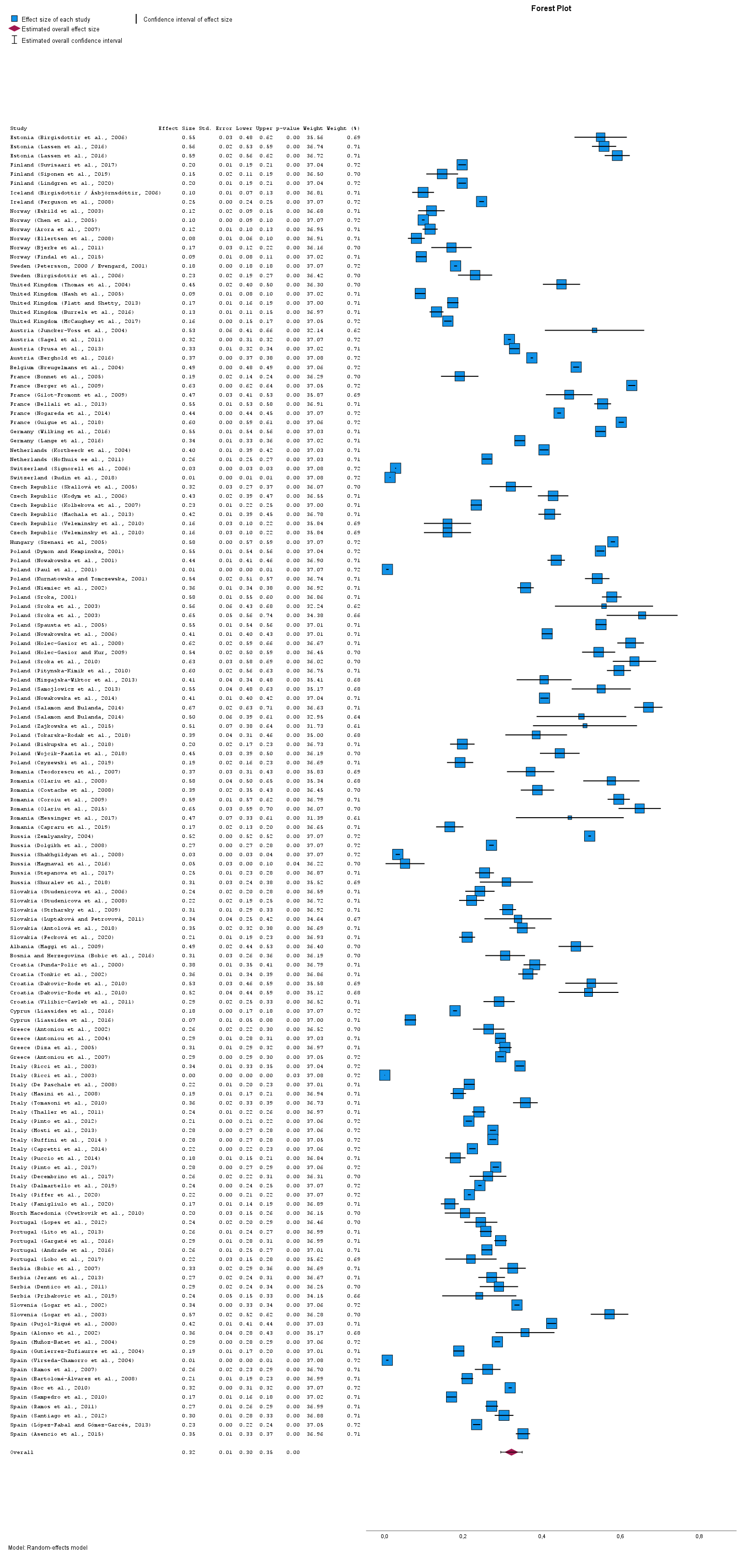

Supplement: Supplementary file 1 [file pathogens-12-01430-s001.zip › Suppl Figure S1.png]

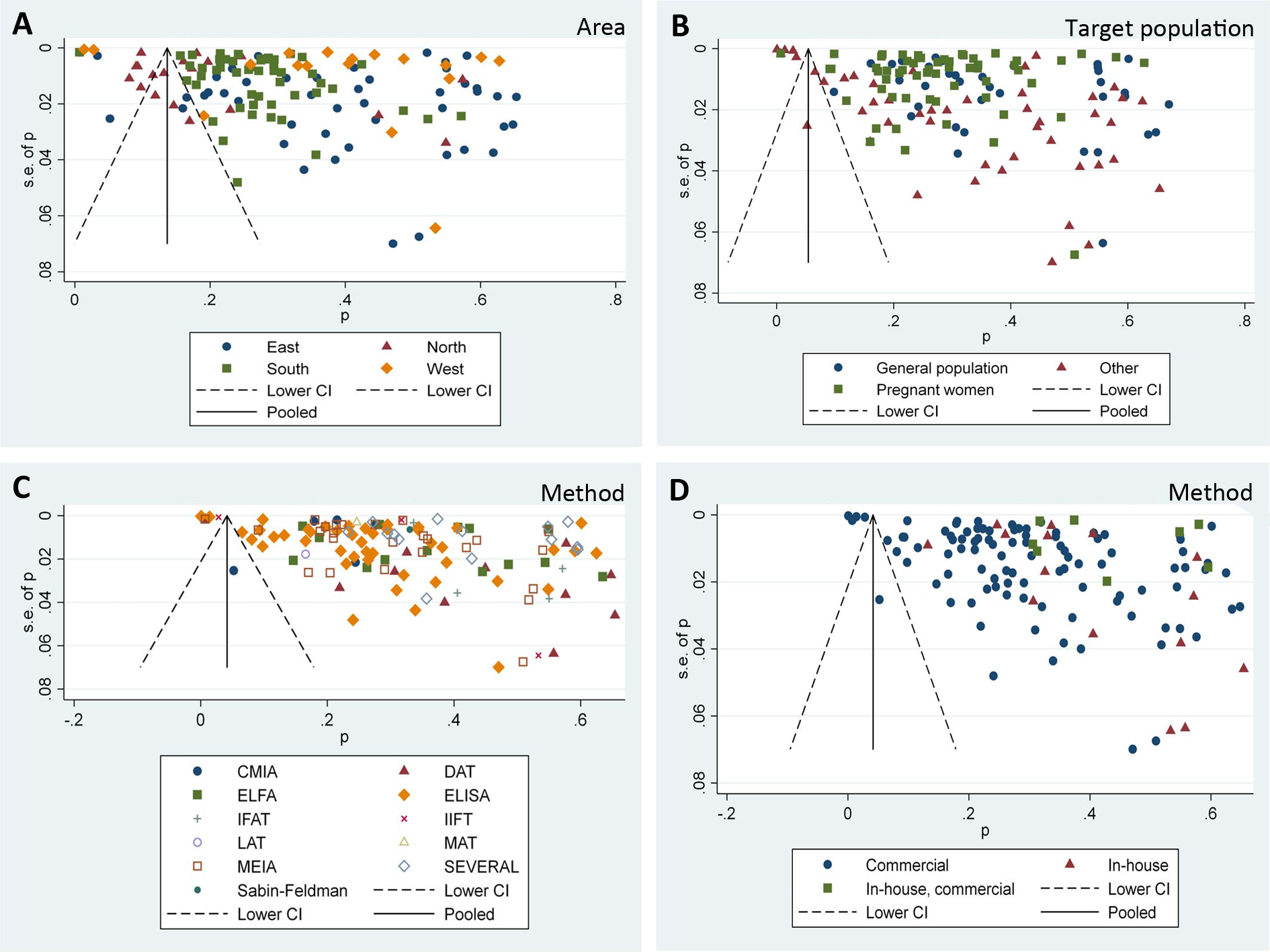

Supplement: Supplementary file 1 [file pathogens-12-01430-s001.zip › Suppl Figure S2.tif]
